# Modulating Direct Growth of Copper Cobaltite Nanostructure on Copper Mesh as a Hierarchical Catalyst of Oxone Activation for Efficient Elimination of Azo Toxicant

**DOI:** 10.3390/nano12244396

**Published:** 2022-12-09

**Authors:** Po-Hsin Mao, Eilhann Kwon, Hou-Chien Chang, Ha Manh Bui, Songkeart Phattarapattamawong, Yu-Chih Tsai, Kun-Yi Andrew Lin, Afshin Ebrahimi, Yeoh Fei Yee, Min-Hao Yuan

**Affiliations:** 1 Department of Environmental Engineering & Innovation and Development Center of Sustainable Agriculture, National Chung Hsing University, Taichung 402, Taiwan; 2 Department of Earth Resources and Environmental Engineering, Hanyang University, SeongDong-Gu, Seoul 133-791, Republic of Korea; 3 Department of Chemical Engineering, National Chung Hsing University, Taichung 402, Taiwan; 4 Department of Environmental Sciences, Saigon University, Ho Chi Minh 700000, Vietnam; 5 Department of Environmental Engineering, King Mongkut’s University of Technology Thonburi, Bangkok 10140, Thailand; 6 Environment Research Center, Department of Environmental Health Engineering, Isfahan University of Medical Sciences Isfahan, Isfahan 81746-73461, Iran; 7 School of Materials and Mineral Resources Engineering, Engineering Campus, Universiti Sains Malaysia, Penang 14300, Malaysia; 8 Department of Occupational Safety and Health, China Medical University, Taichung 40402, Taiwan

**Keywords:** sulfate radical, AOPs, PMS, mesh, catalysts, CuCo_2_O_4_

## Abstract

As cobalt (Co) has been the most useful element for activating Oxone to generate SO_4_^•−^, this study aims to develop a hierarchical catalyst with nanoscale functionality and macroscale convenience by decorating nanoscale Co-based oxides on macroscale supports. Specifically, a facile protocol is proposed by utilizing Cu mesh itself as a Cu source for fabricating CuCo_2_O_4_ on Cu mesh. By changing the dosages of the Co precursor and carbamide, various nanostructures of CuCo_2_O_4_ grown on a Cu mesh can be afforded, including nanoscale needles, flowers, and sheets. Even though the Cu mesh itself can be also transformed to a Cu-Oxide mesh, the growth of CuCo_2_O_4_ on the Cu mesh significantly improves its physical, chemical, and electrochemical properties, making these CuCo_2_O_4_@Cu meshes much more superior catalysts for activating Oxone to degrade the Azo toxicant, Acid Red 27. More interestingly, the flower-like CuCo_2_O_4_@Cu mesh exhibits a higher specific surface area and more superior electrochemical performance, enabling the flower-like CuCo_2_O_4_@Cu mesh to show the highest catalytic activity for Oxone activation to degrade Acid Red 27. The flower-like CuCo_2_O_4_@Cu mesh also exhibits a much lower *E_a_* of Acid Red 27 degradation than the reported catalysts. These results demonstrate that CuCo_2_O_4_@Cu meshes are advantageous heterogeneous catalysts for Oxone activation, and especially, the flower-like CuCo_2_O_4_@Cu mesh appears as the most effective CuCo_2_O_4_@Cu mesh to eliminate the toxic Acid Red 27.

## 1. Introduction

Dyes are extensively employed in the textile, paper, cosmetics, leather, and food industries, and thus effluents from these industries inevitably contain a large amount of dye compounds [1]. Among all types of dyes, azo dyes are the largest group [2]. With the N = N bonding, azo dyes are not only refractory and resistant to biodegradation [3], but also toxic, and sometimes even carcinogenic [4]. Azo dyes can be typically classified into two categories in view of their functional moieties: acidic and basic azo dyes. Particularly, acidic azo dyes (AADs) always receive more attention because AADs have superior water solubilities, and excellent affinities bound to textiles [5]. Among various AADs, Acid Red 27 (AR) represents one of the most consumed AADs with a beautiful reddish color, making it ubiquitous in miscellaneous products. Unfortunately, AR is confirmed as a toxicant, causing allergic and respiratory health issues [6] and even tumors [7]. Therefore, it is highly critical for removing AR from contaminated water to prevent its adverse effects on the environment and public health.

For removing AR, advanced oxidation processes (AOPs) would be a practical technique as AOPs can decompose toxic dyes to lessen their toxicities [8,9,10]. Generally, AOPs are categorized into two types: ^•^OH-related AOPs, and SO_4_^•−^-related AOPs. While several ^•^OH-related AOPs have been proposed for degrading AR, including photocatalysis [11] and the Fenton’s reaction [12], SO_4_^•−^-related AOPs have received increasing interest for degrading AR because SO_4_^•−^ exhibits a higher oxidation power, a higher selectivity for aromatic structures [13], and a longer half-life [14]. 

Oxone (potassium monopersulfate) is a popular commercially available reagent for generating SO_4_^•−^ [15,16]; however, the self-dissociation of Oxone is slow, and therefore “activation” of Oxone is required for the rapid generation of SO_4_^•−^ from Oxone. To date, cobalt (Co) has been confirmed as the most useful element for Oxone activation and a few attempts have been made to evaluate Co_3_O_4_ as a heterogeneous alternative to Co ions for activating Oxone to degrade AR [17,18]. Nonetheless, Co_3_O_4_ nanoparticles (NPs) are prone to aggregate even in water, therefore losing their activities for Oxone activation [19]. Moreover, Co_3_O_4_ NPs and those powder-like Co-based materials are extremely hard to recover after reactions, thereby leading to the secondary pollution. 

Therefore, it would be more practical to decorate nanoscale Co-based oxides on macroscale supports to create a hierarchical catalyst, which would possess both nanoscale functionality and macroscale convenience. Therefore, metal meshes appear as feasible and useful macroscale supports because metal meshes are robust and porous, enabling the growth of nanoscale substances on their surfaces and allowing fluids to flow through easily [20,21]. Among numerous metallic meshes, copper (Cu) meshes (CMs) represent the most accessible metal mesh and CMs can also act as a source of Cu, which is then combined with Co to afford Cu/Co oxides (e.g., CuCo_2_O_4_). Since CuCo_2_O_4_ is comprised of a spinel structure comparable to Co_3_O_4_ and CuCo_2_O_4_ is also considered as a promising catalyst in various reactions [22], the CuCo_2_O_4_ grown on a CM (CCM) should be a promising catalyst for degrading AR through Oxone activation. 

Furthermore, as activities of catalysts would be associated with their appearances, shapes, as well as surface characteristics, CuCo_2_O_4_ with favorable nanostructures on the CM is expected as an excellent catalyst for activating Oxone for eliminating AR. Thus, it would be important and interesting to investigate nanoscale CuCo_2_O_4_ with different morphologies on a CM for optimizing the design of a CCM for efficient Oxone activation to eliminate AR from water. Thus, the present study aims to develop CCMs with various nanoscale morphologies of CuCo_2_O_4_ directly grown on a CM for the first time to investigate the structure–activity relationship of these CCMs via modulating CuCo_2_O_4_ nanostructures. 

## 2. Experimental Section

**Figure 1:**
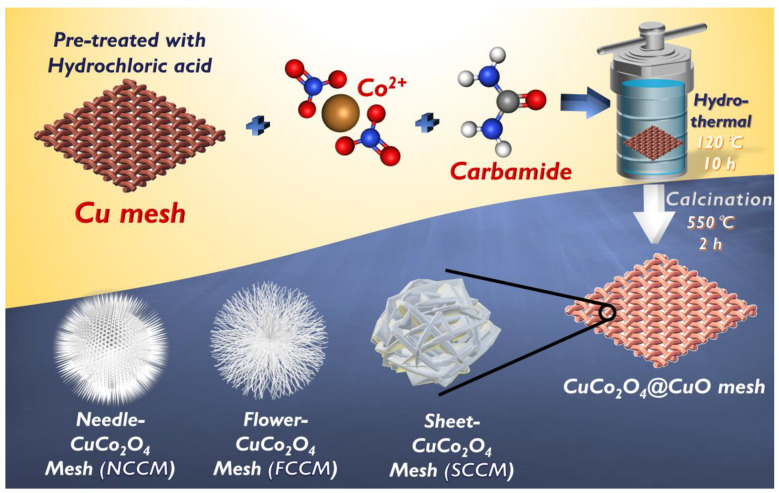
Preparation scheme of NCCM, FCCM, and SCCM.

All reagents used in this study were purchased commercially and employed directly without purification. Co(NO_3_)_2_, carbamide, tert-butyl alcohol (TBA), 5,5-dimethyl-1-pyrroline n-oxide (DMPO), 2,2,6,6-tetramethylpiperidine (TMP), Acid Red 27 (AR), polyvinylidene fluoride, 7,8-Benzoquinoline (BQ), 1-Methyl-2-pyrrolidone, and Oxone were purchased from Sigma-Aldrich (USA). Commercial Co_3_O_4_ NPs (50–80 nm) and sodium azide (NaN_3_) were obtained from Alfa Aesar (USA). Copper mesh was acquired from Maychun Enterprise Co. Ltd. (Taichung, Taiwan). The fabrication of CCMs is depicted in Figure 1 by firstly using the CM as a support and a precursor of Cu, followed by the direct growth of CuCo_2_O_4_ nanostructures hydrothermally with different amounts of Co^2+^ and carbamide. 

A piece of Cu mesh was firstly cut into a rectangular piece (e.g., 20 mm × 20 mm), which was pretreated by cleansing it with the concentrated HCl and rinsing with DI water. Next, 2 mmol of Co(NO_3_) and carbamide (i.e., 2, 4, or 8 mmol) was dissolved in 20 mL of DI water, followed by the addition of the pretreated CM. The resulting mixture was then heated at 120 °C for 10 h, and the corresponding Co-modified CM was then rinsed with DI water, followed by thermal treatment in air for 2 h in 550 °C to produce CuCo_2_O_4_@CM (CCMs). For comparisons, an oxidized Cu mesh (CuOM) was prepared using the aforementioned procedure without the addition of Co(NO_3_) and carbamide. 

The full information of material characterization, AR degradation, analytic methods and computer-aided investigations is provided in the Supplementary Materials. 

## 3. Results and Discussions

### 3.1. Characterization of Catalysts

#### 3.1.1. Morphology and Composition

**Figure 2:**
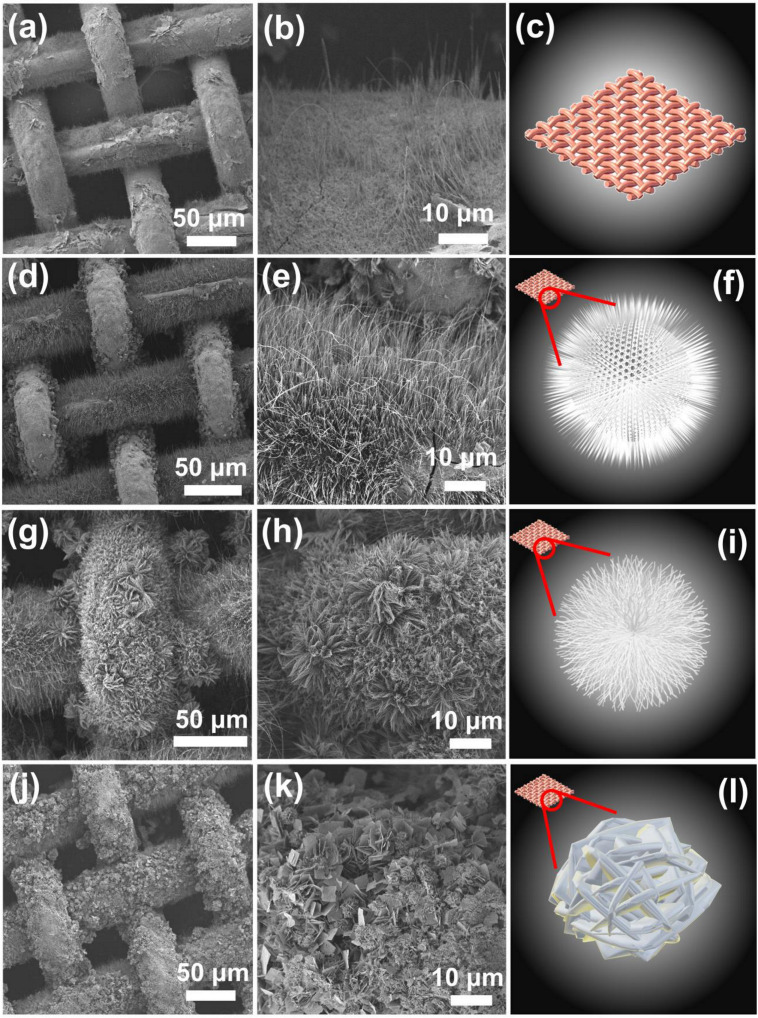
SEM images of (**a**–**c**) CuOM, (**d**–**f**) NCCM, (**g**–**i**) FCCM, (**j**–**l**) SCCM.

**Figure 3:**
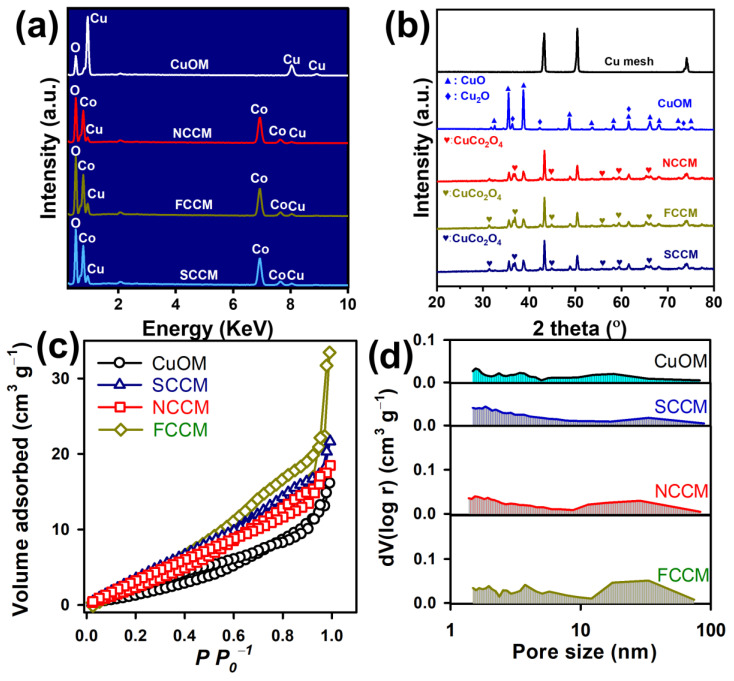
(**a**) EDS, (**b**) XRD patterns, (**c**) N_2_ sorption isotherms, (**d**) pore size distributions of CCMs and CuOM.

As the CM was used as a support, and a source of Cu, the pristine CM was also pretreated, and calcined to afford a Cu-oxidized mesh (CuOM) (Figure 2a) as a reference for comparison with the as-prepared CCMs. In contrast to the pristine CM (as displayed in Figure S1) whose surfaces were smooth, this CuOM showed thorny surfaces covered by numerous tiny thorns. The chemical composition by EDS analysis in Figure 3a unveils that this CuOM consisted of only Cu and O without other elements, indicating that the surfaces on the CM had been oxidized and these thorns might be the Cu-oxide. After Co^2+^ and carbamide were added at a ratio of 1:1 for modifying the CM during the hydrothermal process, its appearance noticeably changed in Figure 2d as the surface of the CM was densely covered by hair-like substances. The closer image (Figure 2e) further displays that those hair-like substances actually consisted of nano-needles on the surface of the CM as illustrated in Figure 2f. Figure 3a also unveils its corresponding chemical composition, in which a noticeable Co signal could be then detected, indicating that these nanoneedles were comprised of Co and Cu.

When the ratio of Co: carbamide was varied to 1:2, there was a resultant modified CM, which is displayed in Figure 2g, suggesting that the mesh structure was preserved but its surface was covered by the flower-like nanostructures. The closer image (Figure 2h) further displays that these flowers were actually assembles of nano-filaments as illustrated in Figure 2i. Figure 3a also shows its corresponding chemical composition, in which a noticeable Co signal can be also found, demonstrating that these flower-like nanostructures were certainly comprised of Co and Cu.

Finally, the molar ratio of Co: carbamide was further changed to 1: 4, and the resultant modified CM is revealed in Figure 2j; the mesh structure remained but its surface was also decorated by many clusters. The closer image (Figure 2k) then unveils that those clusters were comprised of numerous thin sheets which were interpenetrated to assemble as clusters as depicted in Figure 2l. Figure 3a also shows its corresponding chemical composition, in which a noticeable Co signal can be also found, ascertaining that these sheet-like nanostructures also contained Co and Cu.

#### 3.1.2. Crystalline Structures

As these resulting materials all contained Co, Cu, and O, their XRD patterns were determined and are shown in Figure 3b. For comparison, the XRD pattern of the pristine CM was also obtained, and a few peaks can be detected at 43.4, 50.6, as well as 74.6°, attributed to the Cu (111), (200), and (220) planes (JCPDS#03-1018). The CM was oxidized to form a CuOM, and its XRD pattern is unveiled in Figure 3b; the inherent peaks of the CM itself vanished. However, a few additional peaks had emerged, ascribed to Cu oxides, namely, CuO and Cu_2_O. Specifically, the peaks at 29.5, 36.4, 42.3, 61.3, and 73.5° would correspond to the (110), (111), (200), (211), (220), and (311) planes of CuO (JCPDS#900-569), and the peaks at 35.5, 38.8, 48.7, 53.5, 61.5, 65.9, 66.6, 68.1, 72.5, and 75.1° could be ascribed to the (002), (111), (20-1), (020), (11-3), (022), (310), (113), (311), and (004) of Cu_2_O planes (JCPDS#152-6990) (Figure S2). These characterizations demonstrated that the CM surfaces had been converted and oxidized during the pretreatment to afford CuO/Cu_2_O.

After Co^2+^ and carbamide were then introduced, the XRD patterns of these Co-modified CMs preserved multiple CuO/Cu_2_O peaks (Figure 3b). However, several extra peaks would be then detected in all Co-modified CMs, such as the peaks at 31.5, 36.9, 45.1, 56.0, 59.3, 65.7, and 77.0°, attributed to CuCo_2_O_4_ (JCPDS#01-1155), revealing that these nanostructures decorated on CMs could be attributed to CuCo_2_O_4_. These characterizations also unveil that modulating the molar ratio between Co^2+^ and carbamide would shape the morphologies of CuCo_2_O_4_ on the CM distinctly. This was because carbamide would decompose through hydrolysis during the hydrothermal process to generate CO_2_ and NH_3_ [23]. Subsequently, NH_3_ would dissolve in water and become ammonium and a hydroxide ion, which reacts with Co^2+^ and Cu^+^/Cu^2+^ to produce Co/Cu-hydroxides. Simultaneously, NH_4_^+^ then made the reaction solution alkaline, altering rates of precipitation of Co/Cu-hydroxides, and then regulating the morphologic development. The coincidentally resulting CO_2_ would also pressurize the nucleation process of nanocrystals, and influence their shapes [24,25,26]. In general, when a relatively small dosage of carbamide was used, the resulting shapes of CuCo_2_O_4_ would become fibrous, or filament-like as observed in Figure 2. When a relatively high dosage of carbamide was employed, sheet-like shapes of CuCo_2_O_4_ would be afforded as seen in Figure 2j–l [16,27].

To further distinguish these CCMs, the CCM decorated with “needles” is denoted as NCCM, the CCM covered with “flowers” is named as FCCM, and the CCM loaded with clusters of “sheets” is denoted as SCCM.

#### 3.1.3. Textural Properties

As these CCMs interestingly exhibited different morphologies, their textural properties were then determined by N_2_ adsorption in Figure 3c. For comparison, the N_2_ adsorption of the CuOM is also included, and a very limited amount of N_2_ could be adsorbed onto the CuOM; the corresponding isotherm was then classified as a type III model, indicating the weak sorption of N_2_ onto the CuOM, and the porosity was also relatively low. Its pore size distribution (shown in Figure 3d) further validated that a relatively low amount of pores existed in the CuOM, making the CuOM exhibit a relatively low surface area of 10.2 m^2^/g with a total pore volume of 0.024 cc/g. The N_2_ adsorption isotherm of the NCCM is also included in Figure 3c, and its N_2_ adsorption seemed slightly higher than that of the CuOM, possibly owing to the needles decorated on CuM in the NCCM, making the NCCM show a slightly larger specific surface area of 20.3 m^2^/g. Moreover, the pore volume in the NCCM seemed much higher than the CuOM, especially in the range of 2~10 nm with a volume of 0.036 cc/g. Furthermore, the N_2_ adsorption isotherm of the FCCM became much more different from that of the CuOM as a higher N_2_ sorption and a hysteresis loop can be observed, enabling the FCCM to display a larger specific surface area of 29.5 m^2^/g. Its corresponding pore size distribution also validated the presence of mesoscale pores, making the FCCM show a higher total volume of 0.052 cc/g. Moreover, the SCCM seemed to enable moderate N_2_ sorption, making it show a relatively high specific surface area of 24.4 m^2^/g. Its porosity confirmed that the SCCM contained a moderate pore volume, especially from the mesoporous range, leading to a pore volume of 0.032 cc/g. These results also signify that the CuOM possessed a very limited surface area, whereas the Co modification enabled these CCMs to possess much higher surface areas owing to the resultant nanostructures (i.e., needles, flowers, and sheets) for providing more reactive surfaces.

### 3.2. Degradation of AR by Oxone Activation Using Different Catalysts

**Figure 4:**
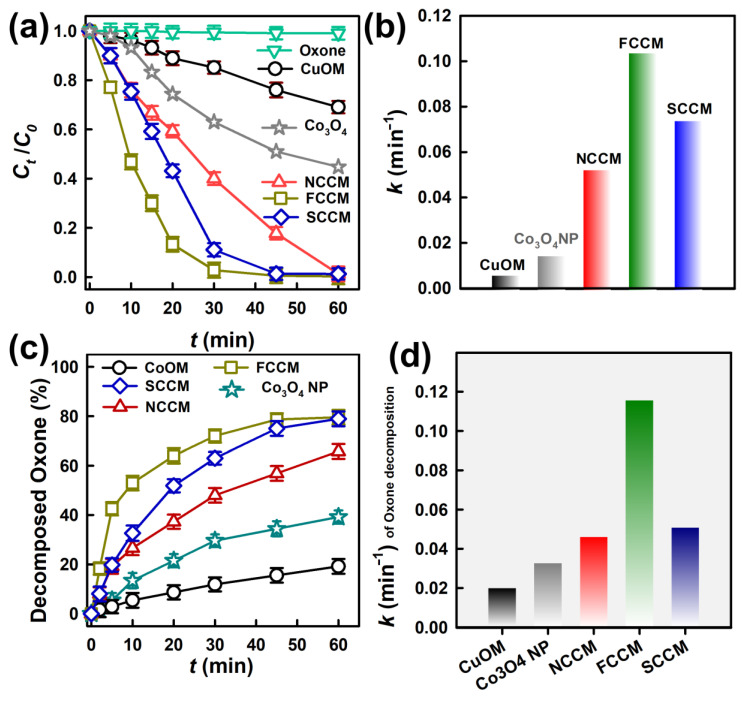
(**a**) Comparison of degradation of AR by Oxone alone, adsorption to catalysts, and Oxone activated by various catalysts, (**b**) decomposition of Oxone by different catalysts, (**c**) decomposed Oxone as a function of time, and (**d**) the kinetics of decomposed Oxone (Catalyst = 200 mg/L, Oxone = 200 mg/L, T = 30 °C).

Firstly, as AR could be eliminated by sorption to catalysts, it would be essential to determine if the AR was removed via sorption to the CCMs. Figure S3 displays that there was no noticeable adsorption of AR onto the CCMs and the CuOM in 60 min, indicating that AR could not be efficiently eliminated via sorption onto the CCMs and the CuOM. Moreover, AR could not be effectively degraded by Oxone alone as shown in Figure 4a, demonstrating that the self-dissociation of Oxone was inefficient and could not degrade AR.

Nevertheless, when the CCMs (i.e., NCCM, FCCM, and SCCM) and Oxone were present together, the concentration of AR decreased rapidly, and *C_t_/C_0_* of AR by these CCMs could all reach zero. These results demonstrate that the CCMs seemed to activate Oxone to cause the degradation of AR. For comparison, the CuOM and Oxone were then introduced to an AR solution; the concentration of AR decreased in 60 min. However, its *C_t_/C_0_* over 60 min only reached 0.69. This result suggests that while the CuOM itself might also activate Oxone to cause the degradation of AR, CuCo_2_O_4_ grown on the CM seemed to accelerate Oxone activation and cause much more rapid AR degradation. On the other hand, since Co_3_O_4_ is a reference catalyst for activating Oxone, a commercially available Co_3_O_4_ nanoparticle (NP) was tested here for comparison. Figure 4a shows that the Co_3_O_4_ NP could also activate Oxone but the corresponding *C_t_/C_0_* in 60 min was merely 0.45. Even though Co_3_O_4_ and CuCo_2_O_4_ both consisted of spinel structures, these CCMs revealed much more superior catalytic activities than the Co_3_O_4_ NP. The hierarchical structure of CCMs might enable the active sites of CuCo_2_O_4_ to expose Oxone more easily, while the Co_3_O_4_ NP actually aggregated seriously, as shown in Figure S4, thereby leading to the relatively low degradation efficiency.

As three CCMs all led to the very rapid degradation of AR, their degradation kinetics were then quantitatively compared using the pseudo-first-order rate law *C_t_ = C_0_ exp*(*-kt*) to determine the rate constant k (min^−1^). The *k* of AR elimination by the NCCM was then calculated as 0.053 min^−1^, the *k* by the FCCM was 0.104 min^−1^, and the *k* by the SCCM was 0.074 min^−1^. In comparison to the *k* values by the CuOM (i.e., 0.006 min^−1^) and Co_3_O_4_ (0.014 min^−1^), three CCMs all showed significantly faster degradation kinetics, validating that the growth of three amounts of CuCo_2_O_4_ on the CM substantially boosted the catalytic activities of Oxone activation. Moreover, among these three CCMs, the FCCM certainly enabled much faster AR degradation kinetics, suggesting that the FCCM possessed a much more advantageous catalytic activity than the NCCM and the SCCM.

As the activation of Oxone involves electron transfer and redox reactions [28], heterogeneously catalytic Oxone activation largely relates to electrochemical properties [29]. Therefore, it would be interesting to further probe into the electrochemical behaviors of these CCMs for distinguishing their different catalytic activities of Oxone activation.

**Figure 5:**
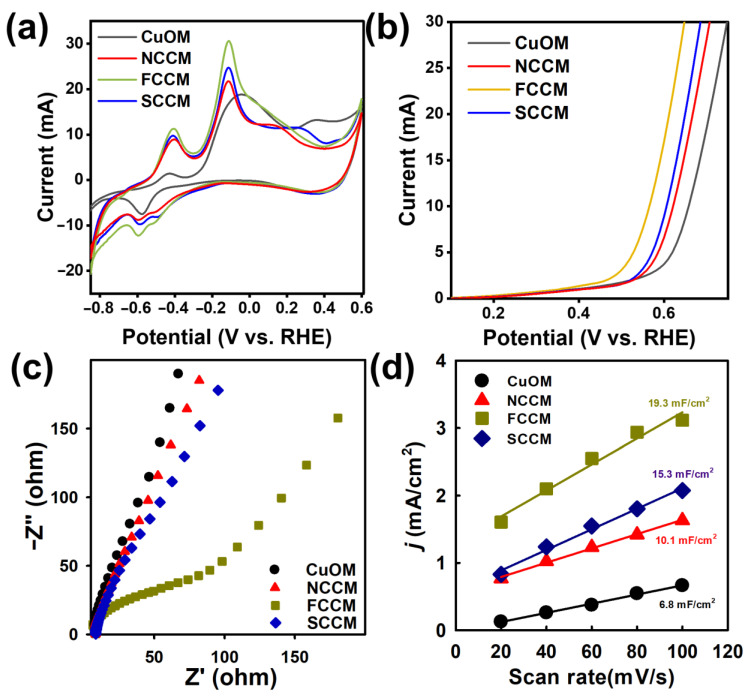
(**a**) CV curves at a scan rate of 60 mV/s, (**b**) LSV curves, (**c**) EIS Nyquist plots, and (**d**) C_DL_ of different catalysts.

Therefore, the electrochemical characteristics of CCMs were firstly measured to probe into their redox potentials and electron transports. Firstly, the cyclic voltammetry (CV) curves of CCMs were obtained and are shown in Figure 5a. The CV curve of the CuOM was also acquired and is shown in Figure 5a as a reference. It exhibits an oxidation peak, possibly attributed to the oxidation of Cu_2_O to CuO, and the two noticeable reduction bands were due to the reduction of Cu^2+^ as well as Cu^+^. In comparison to the CuOM, the CCMs all exhibited much larger current responses than that of the CuOM, indicating that the growth and decoration of CuCo_2_O_4_ on the CM would enhance interfacial reaction rates [30], which would then facilitate the catalytic activation of Oxone. In addition, among these three CCMs, the FCCM exhibited the largest current response, suggesting that the FCCM possessed a much more superior electrochemical performance than the NCCM and the SCCM. This was possible because the FCCM possessed the larger surface area and porosity which acted as ion reservoirs [28,30], decreasing the diffusion distance to the inner surface and accelerating the diffusion process of ions [31]. Moreover, these CCMs also revealed more redox peaks than the CuOM, demonstrating that the CCMs had more electroactive sites, which also improved their catalytic activities.

Next, their corresponding linear sweep voltammogram (LSV) analyses are then displayed in Figure 5b, in which all CCMs showed a much smaller overpotential (at 10 mA) than the CuOM, indicating that the growth of CuCo_2_O_4_ on the CM also enhanced electron transport. Moreover, the FCCM exhibited the smallest overpotential among these three CCMs.

Additionally, the electrochemical impedance spectroscopy (EIS) would be used for assessing the charge transfer ability of CCMs in terms of Nyquist plots as shown in Figure 5c. In comparison to the SCCM and the NCCM, the FCCM exhibited a much shorter diameter of the semi-circle at the high-frequency region, signifying a smaller charge transfer resistance, and also a much more rapid electron transfer rate in the FCCM [32], enabling the FCCM as a more efficient CCM for Oxone activation.

On the other hand, for further comparing the active sites of these catalysts, the scan-rate-dependent CV curves of these CCMs and the CuOM in the non-Faradaic region with 1.0M of KOH were obtained, and are shown in Figure S5a–d. The double-layer capacitance (C_DL_) was then computed using C_DL_ = *J_m_*/*v*, where *J_m_* represents the current density and *v* means the scanning rate. *J_m_* could be then calculated by averaging values between the anodic and cathodic current densities with the central potential by $J_{m}=\left(\left|J_{a}\right|+\left|J_{c}\right|\right)/2$. Via the linear regression of *J_m_* with v, the slopes of the fitting lines afforded C_DL_ values of these CCMs and the CuOM as shown in Figure 5d. The FCCM exhibited a significantly higher C_DL_ value of 19.3 mF/cm^2^, followed by the SCCM (15.3 mF/cm^2^), then the NCCM (10.1 mF/cm^2^), and finally the CuOM (6.8 mF/cm^2^). These comparisons suggested that the FCCM exhibited the highest area of active sites among all these catalysts. These results all indicated that the FCCM possessed the much more superior electrochemical properties, thereby exhibiting significantly higher catalytic activity towards Oxone activation, and leading to the fastest rate constant. These analyses ascertained the growth of CuCo_2_O_4_ on the CM would significantly improve their electrochemical properties, making these CCMs promising catalysts for Oxone activation.

### 3.3. Effects of Oxone and Catalyst Dosages

**Figure 6:**
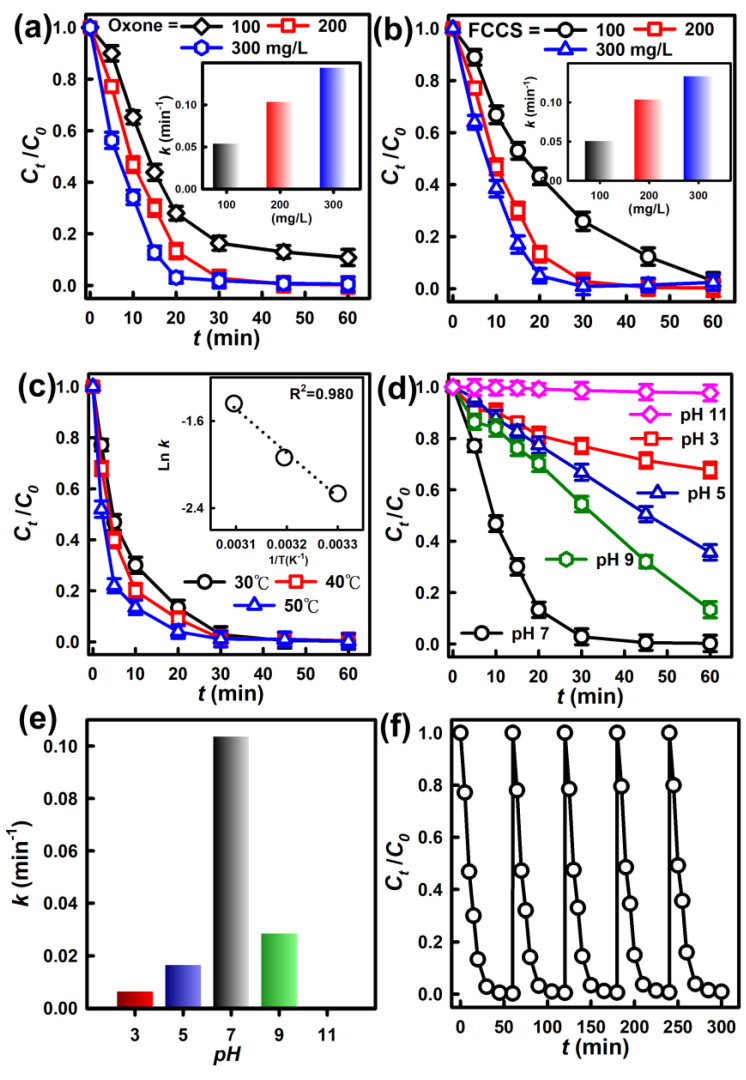
Effects of (**a**) catalyst, (**b**) Oxone, (**c**) temperature, (**d**,**e**) pH on AR degradation and (**f**) recyclability (catalyst = 200 mg/L; Oxone = 200 mg/L; T = 30 °C).

While the FCCM was found to efficiently eliminate AR via activating Oxone, it was useful to further assess the effects of Oxone and FCCM dosages for further realizing their respective effects. Figure 6a first displays AR degradation by varying Oxone from 100 to 300 mg/L with a constant dosage of FCCM as 200 mg/L. AR elimination was greatly influenced at different Oxone dosages as a lower Oxone dosage of 100 mg/L led to much slower degradation, and its corresponding *C_t_/C_0_* merely achieved 0.11 with a smaller *k* of 0.054 min^−1^. Once Oxone became 200 mg/L, the AR degradation would be slightly enhanced as its *C_t_/C_0_* reached zero quickly with a k of 0.104 min^−1^. In the case of Oxone = 300 mg/L, the AR could be completely eliminated at an even shorter time with a higher *k* of 0.144 min^−1^. This demonstrates that the dosage of Oxone was critical because the availability of Oxone greatly influences the amounts of reactive oxygen species (ROS).

**Figure 7:**
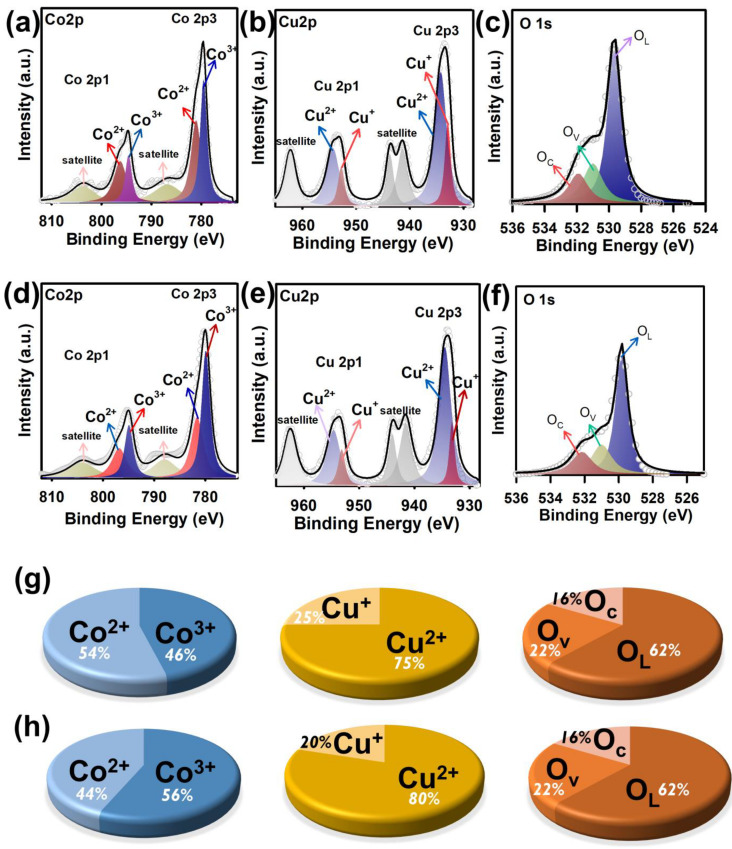
XPS analyses: (**a**–**c**) pristine FCCM, and (**d**–**f**) used FCCM; fractions of species in the (**g**) pristine and (**h**) spent FCCM.

On the other hand, when the FCCM changed from 200 to 100 mg/L, the AR could be still degraded completely in 60 min. Nevertheless, a relatively low FCCM dosage obviously caused the degradation to proceed more slowly as indicated in the insets of Figure 7b. Once the FCCM dosage was then raised from 200 to 300 mg/L, the AR degradation was accelerated, reaching the complete elimination within a shorter time at a *k* = 0.133 min^−1^. Such a phenomenon has been also observed in several studies of using Oxone to degrade contaminants because a higher dosage of catalyst would provide more reactive surface areas of catalysts, which would facilitate Oxone activation, and then enhance degradation [33]. These results also demonstrated that, even with a relatively low dosage of 100 mg/L, the FCCM still led to very quick and effective AR degradation, suggesting that the growth of CuCo_2_O_4_ on the CM had enabled the CM to be a highly efficient catalyst for Oxone activation.

### 3.4. Other Effects and Reusability

#### 3.4.1. Variation in Temperature

For further investigating the catalytic behaviors of the FCCM, AR degradation was then implemented at various temperatures (30, 40, and 50 °C) in Figure 6c. In general, AR degradation was considerably accelerated at higher temperatures by using the FCCM. For instance, the FCCM at 30 °C could rapidly degrade AR and *C_t_/C_0_* could approach zero at 60 min with a *k* of 0.104 min^−1^, then increase to 0.144 min^−1^ at 40 °C, and 0.237 min^−1^ at 50 °C. This indicates the positive effect of the higher temperature on AR elimination.

Moreover, since the *k* increased along with higher temperatures, the correlation of *k* with *T* was then associated with each other for obtaining *E_a_* by the following the Arrhenius equation:

Ln*k* − lnA = −*E_a_*/RT

The inset of Figure 6c further displays a plot of *ln k* vs. *1/T*, which is fitted linearly with R^2^ >0.980, indicating that the relationship of *k* vs. *T* was correlated via the Arrhenius equation. The *E_a_* value of AR degradation by FCCM-activated Oxone was calculated as 33.5 kJ/mol, which was smaller than most of the reported *E_a_* of AR degradation by other processes, ranging from 39 to 70 kJ/mol, in Table S1 [34,35,36,37,38,39,40,41,42,43,44], thereby confirming that the FCCM was certainly an advantageous and highly useful catalyst for degrading AR.

#### 3.4.2. Effect of pH

Furthermore, it would be essential to investigate how pH affected the AR elimination. Figure 6d displays that the AR was rapidly eliminated at pH = 7 with *k* = 0.104 min^−1^. As the pH was adjusted to 5, the AR elimination was slightly influenced with *C_t_/C_0_* = 0.35 and a *k* of 0.016 min^−1^. Such a negative influence was more obvious at pH = 3, *C_t_/C_0_* only achieved 0.67, and the *k* dropped to 0.006 min^−1^. AR elimination by the FCCM was also affected substantially under basic conditions. For instance, AR elimination at pH = 9 was lightly affected as *C_t_/C_0_* still achieved 0.13 at 60 min, and its *k* decreased slightly to 0.029 min^−1^. Once the pH rose to 11, a highly alkaline condition, the AR was barely degraded with a significantly small *k* of 0.007 min^−1^.

Under acidic conditions, Oxone is shown to remain relatively indolent [45], thereby making Oxone activation relatively inefficient. Moreover, ROS generated from Oxone might be consumed by H^+^ through potential reactions as follows [46], causing less efficient AR elimination.

H^+^ + ^•^OH + e^−^ → H_2_O

H^+^ + SO_4_^•−^ + e^−^ → HSO_4_^−^

Moreover, as *pK_a_* of AR is 6.5 [47], AR tends to show positive charges under low-pH conditions. Therefore, the less efficient elimination of AR at pH = 3 was possibly ascribed to the more intense revulsion between these CCMs and AR at lower pH. Simultaneously, H^+^ would also interact with radicals at low pH [48], thereby diminishing the efficiency of Oxone for AR elimination. Hydrogen bonding between O-O/proton on Oxone itself at low pH is also reported to limit the interactions between Oxone and catalysts [49].

Furthermore, the highly basic condition would be unfavorable for AR elimination because Oxone would become SO_5_^2−^ under the basic condition as the *pK_a_* of Oxone is 9.4 (Figure S6). Thus, the revulsion between the FCCM and SO_5_^2−^ would be strengthened, prohibiting the production of ROS from Oxone [19].

#### 3.4.3. Reusability of the FCCM and the Activation Mechanism of the FCCM for Oxone

In addition to the effects of temperature and pH, the reusability of a heterogeneous catalyst is also crucial. Therefore, the reusability of the FCCM was then examined by using the FCCM over five consecutive cycles in Figure 6f. Figure 6f reveals that the spent FCCM was still be capable of activating Oxone to degrade AR, and no significant loss of catalytic activity could be observed, demonstrating the high reusability of the FCCM. In general, the number of tests for the recyclability evaluation of a heterogeneous catalyst in AOPs typically ranges from 3 to 6 [10,50]. Thus, five cycles of AR degradation by the FCCM were conducted. While the FCCM still remained active for activating Oxone to degrade AR during the recyclability test, the AR degradation was slightly influenced as the degradation kinetics seemed slightly slower. This phenomenon might be attributed to the accumulation of degradation by-products on the surface of the FCCM, hindering the reaction between the active site and Oxone. This issue might be resolved by calcining the catalyst again to eradicate the impurities present on the catalyst.

Moreover, the surface chemistry of the FCCM using X-ray photoelectron spectroscopy before and after the recyclability was analyzed, and the core-level Co, Cu, and O spectra of the FCCM were measured and are displayed in Figure 7a–c, respectively. Specifically, the Co2p spectrum of the pristine FCCM can be then deconvoluted to reveal a series of notable peaks at 778.2, 780.6, 795.4, and 796.8 eV. These peaks at 778.2 as well as 795.4 eV could be related to Co^3+^, whereas the peaks at 780.6 and 796.8 were assigned to Co^2+^. Moreover, the Cu2p spectrum could be deconvoluted to result in numerous peaks at 933.5 and 953.4 eV, corresponding to Cu^3+^, and the peaks at 939.4, 943.3, 959.2, and 962.7 eV attributed to the Cu^3+^ satellite peaks. In addition, its O1s spectrum was deconvoluted into several bands at 529.3, 530.5, as well as 531.7 eV as shown in Figure 7c, ascribed to O_lattice_ (O_L_), O_vacancy_ (O_V_), and O_chemi-sorbed_ (O_C_). O_V_ is proven to relate to catalytic activities of Oxone activation [24]. On the other hand, the spent FCCM had also been analyzed, and its Co, Cu, and O core-level spectra are displayed in Figure 7d–f, respectively. Essentially, these core-level spectra of the spent FCCM were very comparable to those spectra of the pristine FCCM, suggesting that Oxone activation and AR degradation did not cause significant changes in the FCCM. Nevertheless, the species ratios of Co and Cu before and after the recyclability test slightly varied as shown Figure 7g,h. The fraction of Co^2+^ seemed smaller in the spent FCCM, whereas the fraction of Cu^+^ also became relatively low in the spent FCCM. These variations were consistent with the reported mechanism of Oxone activation, in which metal species mediate Oxone activation through the transformation of valences of metal species as follows [51,52]:

M^+1^ + HSO_5_^−^ → M^+2^ + SO_4_^•^^−^ + OH ^−^

M^+2^ + HSO_5_^−^ → M^+1^ + SO_5_^•^^−^ + OH ^−^

Thus, these results also validated that AR degradation by FCCM-activated Oxone might be attributed to the redox process of metal species in the FCCM (i.e., Co/Cu). Moreover, the fraction of O_V_ in the FCCM before and after the recyclability did not significantly change, suggesting that O_V_ might not be the principal factor contributing to the catalytic activity of the FCCM for activating Oxone.

### 3.5. Mechanistic Insights into AR Elimination

**Figure 8:**
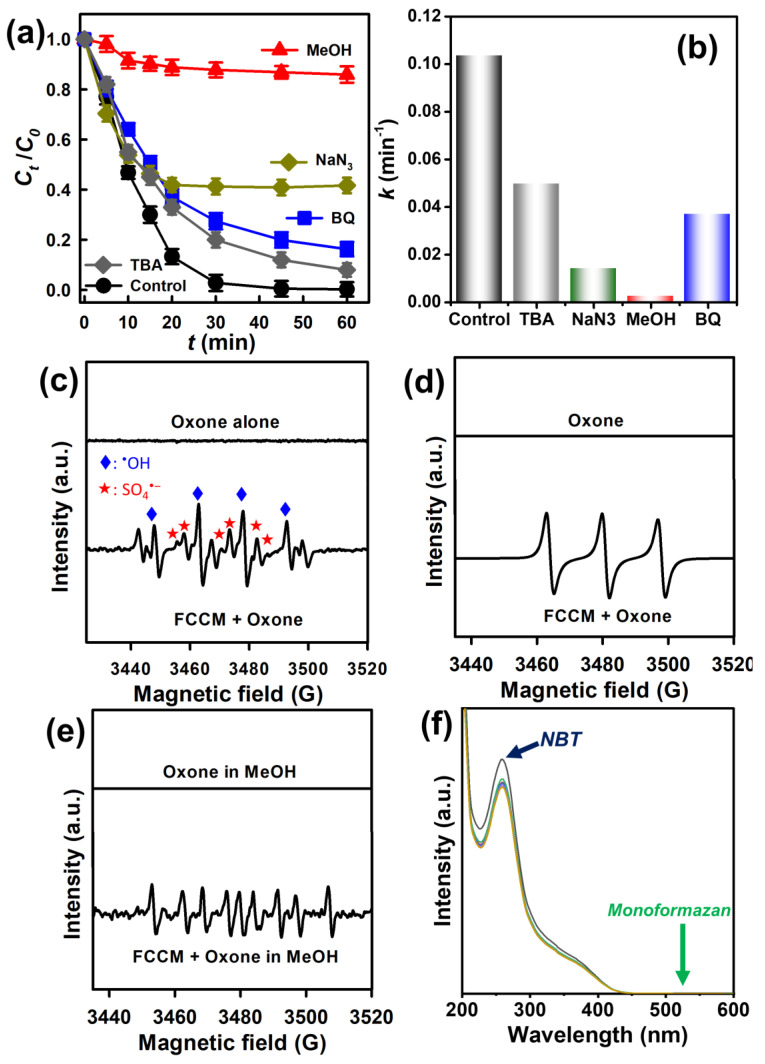
Effects of radical probe on the degradation of AR by the FCCM: (**a**) degradation results, (**b**) rate constants. EPR analyses: (**c**) DMPO, (**d**) TMP, (**e**) DMPO in methanol, and (**f**) NBT test for superoxide (catalyst = 200 mg/L; MPS = 200 mg/L; T = 30 °C).

For further studying the mechanism for Oxone activation by the FCCM in AR degradation, a few radical probes were used to elucidate ROS produced from FCCM-activated Oxone. Initially, tert-butanol (TBA), containing no *α*-hydrogen, acted as a radical probe of ^•^OH. Figure 8a displays that the addition of TBA caused a slight interference in AR degradation by the FCCM as the corresponding *k* became much smaller from originally 0.104 to 0.050 min^−1^ (Figure 8b), demonstrating that ^•^OH might be present during AR degradation.

On the other hand, methanol was utilized to act as a radical probe for both of ^•^OH and ${\mathrm{SO}}_{4}^{•-}$. Figure 8a displays that AR elimination was considerably suppressed as the corresponding *k* merely reached 0.002 min^−1^ in the presence of methanol. Next, NaN_3_ was selected as a probe for detecting the existence of ^1^O_2_ as Oxone is validated to induce the formation of a nonradical ROS, singlet oxygen (^1^O_2_) [53]. Figure 8b reveals that AR degradation by the FCCM was hugely affected because *k* decreased to 0.014 min^−1^, and thus ^1^O_2_ might be involved in AR elimination. On the other hand, 7,8-Benzoquinoline (BQ), a probe for superoxide, O_2_^•–^, was also added during AR degradation, which was noticeably influenced as the corresponding k decreased to 0.037 min^−1^. These results demonstrate that TBA, BQ, and NaN_3_ noticeably influenced AR degradation, while methanol caused an even more significant inhibition to AR degradation. Therefore, these results suggest that ${\mathrm{SO}}_{4}^{•-}$,^•^OH, O_2_^•–^, and ^1^O_2_ might exist in FCCM-activated Oxone and contribute to AR degradation.

Furthermore, electron paramagnetic resonance (EPR) spectroscopy was then adopted to examine ROS produced from FCCM-activated Oxone. Firstly, 5,5-Dimethyl-1-pyrroline N-Oxide (DMPO) was adopted as the spin-trapping agent; almost no signal was detected using DMPO and Oxone (Figure 8c). After DMPO, the FCCM and Oxone were concurrently added, and the notable signals of DMPO-SO_4_ and DMPO-OH were observed, validating the presence of ${\mathrm{SO}}_{4}^{•-}$ and ^•^OH.

Next, 2,2,6,6 Tetramethylpiperidine (TMP) was then adopted as the spin-trapping agent for examining the presence of ^1^O_2_. As no notable pattern was obtained by Oxone alone (Figure 8d), a noticeable triplet pattern was then detected in the mixture of TMP, Oxone, and the FCCM, corresponding to TMPO, ascertaining the existence of ^1^O_2_ generated from Oxone activation. Nevertheless, when the DMPO, Oxone, and FCCM were simultaneously added to methanol, the corresponding EPR result (Figure 8e) displayed 5,5-dimethyl-2-pyrrolidone-N-oxyl (DMPOX) instead of DMPO-O_2_^•−^. For further elucidating if O_2_^•−^ occurred and was derived from the FCCM-activated Oxone, another method of using nitro blue tetrazolium chloride (NBT) was also adopted because NBT has been also a long-lasting probe for determining the presence of O_2_^•−^ which converts NBT to monoformazan. Nonetheless, no obvious peak of monoformazan at 530 nm could be detected (Figure 8f), suggesting that the presence of O_2_^•−^ might be negligible. However, the inhibitory effect of BQ observed in Figure 8a might be attributed to the accelerated dissociation of Oxone by the presence of BQ [54], as well as the competitive adsorption between BQ and the catalytic sites of the FCCM. Therefore, major species contributing to AR degradation by the FCCM-activated Oxone could be ascribed to SO_4_^•−^, ^•^OH, and ^1^O_2_.

### 3.6. Computational Calculation and Possible AR Degradation Pathways and

**Figure 9:**
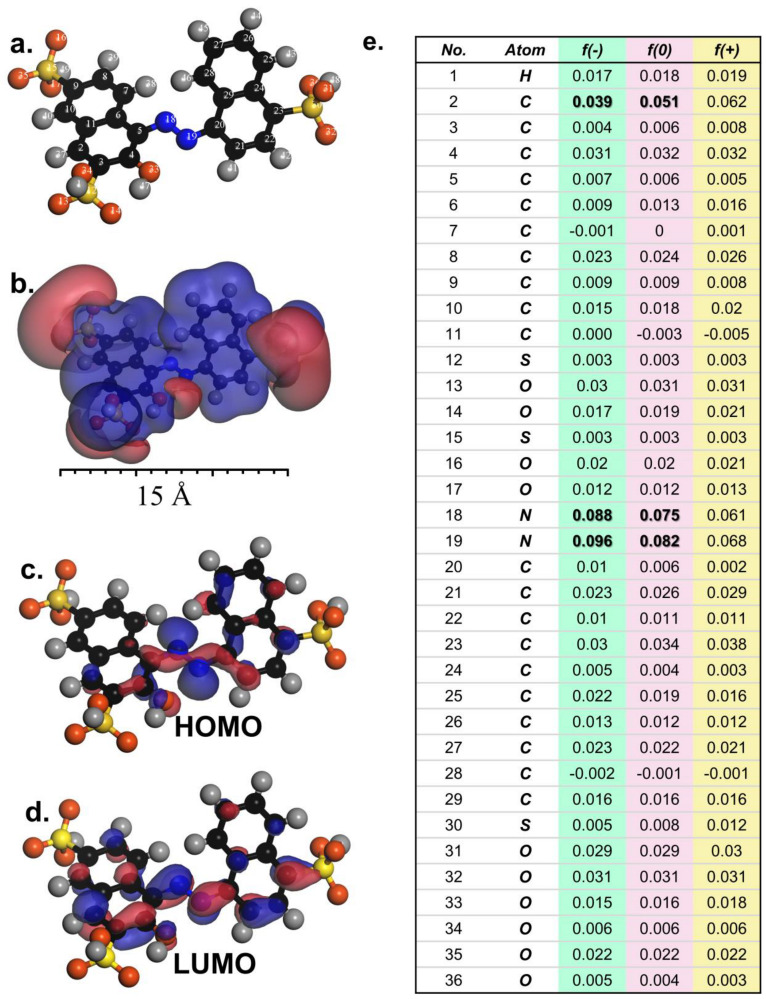
DFT calculation for AR: (**a**) the optimized molecule structure; (**b**) electrostatic potential (ESP); (**c**) HOMO; (**d**) LUMO; and (**e**) condensed Fukui index distribution for electrophilic attack (f^–^), radical attack (f^0^), and nucleophilic attack (f^+^).

As AR could be effectively degraded using Oxone activated by the FCCM, it was interesting to further study the decomposition route of AR. To this end, density functional theory (DFT) computation would then offer insights into the susceptibilities of reactive sites of AR according to the molecular orbitals (MOs) and Fukui indices of AR [55,56,57]. Figure 9a,b show the optimized structure with atomic labels and electrostatic potential (ESP)-mapped configuration of AR, in which the red-colored and blue-colored regions represent the electron-abundant and electron-scarce parts of the AR.

Those electron-abundant parts might attract electrophilic attacks, especially by ^•^OH and SO_4_^•−^. On the other hand, Figure 9c,d display the highest occupied MO (HOMO) and lowest unoccupied MO (LUMO), respectively. Specifically, the HOMO on the azo bonding (i.e., N = N) might tend to release electrons; therefore, the decomposition of AR might be initiated by assaults on the azo group with the electrophilic ROS.

Moreover, the Fukui indices of AR were also calculated for predicting the most possible reaction sites of AR, as summarized in Figure 9e with lists of *f* ^–^, *f* ^0^, and *f* ^+^. In general, a site with a higher *f* ^–^ index tended to accept electrophilic attacks, and N18 and N19 exhibited much higher *f* ^–^ indices than any other sites, demonstrating that these sites were the most possible sites for drawing electrophilic assaults. This result was in line with the aforementioned observation that the azo group of AR might be easily attacked. Moreover, in addition to the azo group, C2 showed a relatively higher *f* ^–^ value than other carbon sites, suggesting that AR might also receive attacks on its carbonaceous structure, possibly through ring-opening reactions. On the other hand, the list of *f* ^0^ also suggests that N18 and N19 (the azo group) might also attract the so-called “non-radical” attacks, possibly from the singlet oxygen.

**Figure 10:**
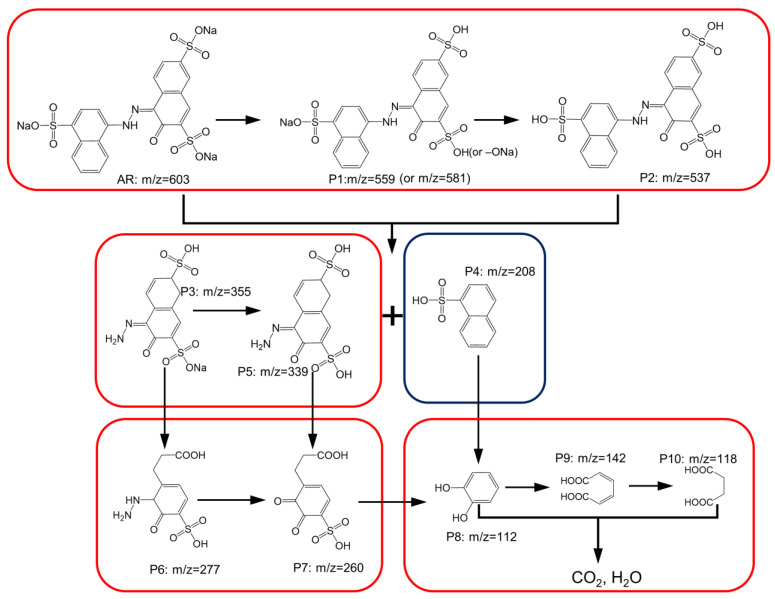
A proposed degradation process of AR by FCCM-activated Oxone based on the detected intermediates.

For further probing into the decomposition route of AR, the decomposition of AR by Oxone activated using the FCCM was then monitored using mass spectrometry as displayed in Figure S7, and the potential degradation intermediates are listed in Table S2. In view of these intermediates and the understanding obtained from the DFT calculation, a possible decomposition route for AR by the FCCM-activated Oxone could be interpreted, as illustrated in Figure 10. Initially, AR (*m*/*z* = 603) might have transferred to its analogous intermediates after the group of sodium sulfate was replaced by the hydroxyl group, affording P1 and P2. Next, AR, P1, or P2 were then further decomposed through the cleavage of the azo group, generating a series of by-products, such as P3 (which might evolve into P5) and P4. Subsequently, P3 (and P5) might also have been oxidized to open rings and eliminated the sulfate group to become P6 (and P7). Simultaneously, P4 might then have been decomposed via the ring-opening reaction to afford P8, which might also have been derived from the decomposition of P7. Afterward, the resulting P8 would have been continuously oxidized to generate even smaller molecules, such as P9 and P10, and eventually CO_2_ and H_2_O.

## 4. Conclusions

In this study, a facile protocol was proposed for fabricating CuCo_2_O_4_ on a CM by utilizing the CM itself as a Cu source. Through varying dosages of a Co precursor and carbamide, different nanostructures of CuCo_2_O_4_ were successfully created and decorated (grown) on the CM, including nanoscale needles, flowers, and sheets. While the CM itself could also be transformed to grow nanoneedles of Cu-O on its surfaces, becoming a CuOM, the growth of CuCo_2_O_4_ on the CM significantly improved the physical, chemical, as well as electrochemical properties, enabling these CCMs to be much more superior catalysts for activating Oxone to degrade the Azo toxicant, AR. More interestingly, among the NCCM, FCCM, and SCCM, the FCCM exhibited a higher specific surface area and more superior electrochemical performance, enabling the FCCM to show the highest catalytic activity for Oxone activation to degrade AR among these CCMs. The FCCM also exhibited a much lower *E_a_* of AR degradation than the reported catalysts. Moreover, the degradation mechanism of AR by FCCM-activated Oxone was also elucidated using the tests of radical probes and EPR for identifying the presence and contribution of ROS to AR degradation. The AR degradation mechanism could be elucidated, and the degradation pathway was also investigated and unveiled in detail via the DFT calculation. These results validate that CCMs are useful heterogeneous catalysts for Oxone activation, and especially, FCCM appears as the most favorable CCM to eliminate the toxic AR.

## Data Availability

The study did not report any data.

## Supplementary Materials

The supplementary material can be downloaded at: https://www.mdpi.com/2079-4991/12/24/4396/s1. Text S1. Preparation, characterization and analytic methods. Table S1. A comparison of *E_a_* values for AR degradation by Oxone activated by various catalysts. Table S2. Detected by-products of AR degradation by FCCM+Oxone; Figure S1. SEM images of the pristine Cu mesh under different magnifications; Figure S2. Simulated XRD patterns of CuO, Cu_2_O and CuCo_2_O_4_. Figure S3. Removal of AR through adsorption to those catalysts (Catalyst = 200 mg/L, 30 °C). Figure S4. (a) SEM image and (b) Textural properties of the commercial Co_3_O_4_ NP. Figure S5. CV curves of (a) CuOM, (b) NCCM, (c) SCCM, and (d) FCCM at different scan rates. Figure S6. Species of Oxone at various pH; Figure S7. ESI mass spectrum of intermediates of AR degradation.
