# Supplementary material for: Modulating Direct Growth of Copper Cobaltite Nanostructure on Copper Mesh as a Hierarchical Catalyst of Oxone Activation for Efficient Elimination of Azo Toxicant
Source: Nanomaterials (Basel). 2022 Dec 9;12(24):4396. doi: 10.3390/nano12244396 (PMC9853330; doi:10.3390/nano12244396)
Supplement: Supplementary file 1 [file nanomaterials-12-04396-s001.zip › nanomaterials-2008204-supplementary.pdf]

Electronic Supporting Information

for

Modulating Direct Growth of Copper Cobaltite Nanostructure on Copper Mesh as a Hierarchical Catalyst of Oxone Activation for Efficient Elimination of Azo Toxicant

Text S1. Preparation, characterization and analytic methods.

Characterization of catalysts

The appearance of all materials was determined using SEM and TEM (JEOL, Japan). Moreover, the crystalline structures of all as-prepared materials were characterized using an X-ray diffractometer (Bruker, USA). The surface chemistry of catalysts was further examined using X-ray photoelectron spectroscopy (XPS) (ULVAC-PHI, PHI 5000, Japan). The BET surface area and pore volume of catalysts were determined by N_2 sorption isotherms using a volumetric analyzer (Anton Paar Autosorb IQ, Austria).

The electrochemical analysis was measured on an electrochemical workstation at room temperature using a standard three-electrode cell. A Pt wire as the counter electrode, an Ag/AgCl electrode as the reference electrode, and 20 μl of active suspension dropping on the glass carbon electrode was used as the working electrode. Dispersed 16 mg of catalysts, 2 mg of carbon black and 2 mg of polyvinylidene fluoride in 10 ml of 1-Methyl-2-pyrrolidone to form an active suspension. The electrolyte that can offer ions and assure the reversible chemical reaction was selected 1M KOH (pH 13) or 0.5M Na_2SO_4 (pH 6.6).

The cyclic voltammetry (CV) was operated at a scan rate of 60 mV s^{-1} in 1 M KOH solution. Using the 10mV scan rate in 1 M KOH electrolyte to obtain the linear sweep voltammogram (LSV) curves. Appling a frequency from 10 Hz to 10000 Hz with a 5 mV amplitude to detect the electrochemical impedance spectroscopy (EIS).

Degradation of AR

The degradation of AR using Oxone was conducted using batch-type experiments. Typically, a certain amount of catalyst (i.e., 40 mg) was firstly added into 200 mL of AR solution with the initial concentration (C_0) of AR of 5 mg/L. Next, 40 mg of Oxone was instantly introduced to the AR solution to start the degradation experiment. At a certain reaction time (t), sample aliquots were withdrawn and filtered to separate catalysts from the AR solution. The remaining AR concentration in the filtrate at t min (C_t) was subsequently determined by HPLC with a UV-Vis detector at 568 nm.

The reactive oxygen species (ROS) involving in the degradation of AR were revealed by electron paramagnetic resonance (EPR) with a typical radical spin-trapping agent, namely 5,5-Dimethyl-1-pyrroline N-oxide (DMPO), and 2,2,6,6-tetramethylpiperidine (TMP). The recyclability of catalysts for multiple AR

degradation cycles was also evaluated by re-using catalysts. Besides, the degradation intermediates produced from AR degradation were further identified using a mass spectrometer (Thermo Finnigan Corporation, LCQ ion-trap mass spectrometer, USA).

DFT calculation

Active sites on the AR molecule and intermediates can be realized through Fukui function using DFT calculation which was performed with Gaussian 16 software: method = B3LYP and basis set = 6-31+g(d,p).

Definition of Fukui function and Fukui indexes (f^-, f^0, f^+)

Fukui function: $f(r) = \left[\frac{\partial \rho(r)}{\partial N}\right]_v$

Where; ρ(r) is the electron density at a point γ in space; N is the electron number in the system; v is the external potential.

Fukui function (f) in reaction:

Nucleophilic attack: $f^+(r) = \rho_{N+1}(r) - \rho_N(r) \approx \rho^{LUMO}(r)$

Electrophilic attack: $f^-(r) = \rho_N(r) - \rho_{N-1}(r) \approx \rho^{HOMO}(r)$

Radical attack:

$$f^0(r) = \frac{f^+(r) + f^-(r)}{2} = \frac{\rho_{N+1}(r) - \rho_{N-1}(r)}{2} \approx \frac{\rho^{HOMO}(r) + \rho^{LUMO}(r)}{2}$$

Table S1. A comparison of E_a values for AR degradation by Oxone activated by various catalysts.

Catalyst	E_a (kJ/mol)	Reference
FCCM	33.5	This work
CoNSC	39.4	[1]
MCC	64.5	[2]
$CoTiO_3$	52.1	[3]
CoCNF	70.4	[4]
CF@CNF	35.8	[5]
Co@CN	43.1	[6]
CoBC	48	[7]
CONF	43.5	[8]
CF@SNC	53	[9]
Co@MS	39.8	[10]
Ferrocene	62.9	[11]

Table S2. Detected by-products of AR degradation by FCCM+Oxone

	Structure/Name/Chemical Formula	m/z
AR	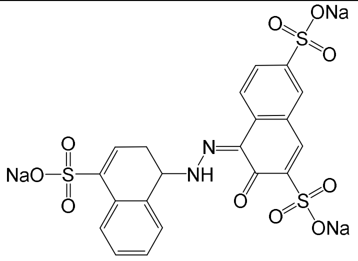 Chemical Formula: $C_{20}H_{13}N_2Na_3O_{10}S_3$	603
P1	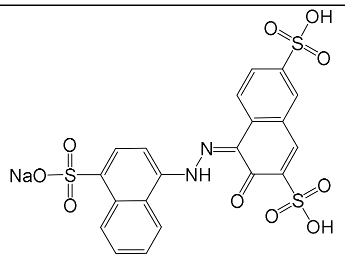 Chemical Formula: $C_{20}H_{13}N_2NaO_{10}S_3$	559
P2	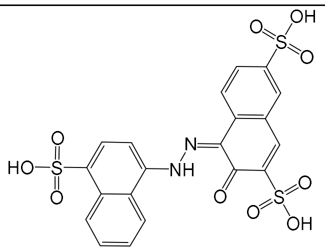 Chemical Formula: $C_{20}H_{14}N_2O_{10}S_3$	537
P3	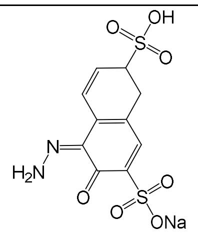 Chemical Formula: $C_{10}H_9N_2NaO_7S_2$	355
P4	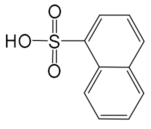 Chemical Formula: $C_{10}H_8O_3S$	208

P5	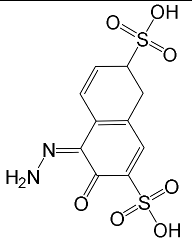 Chemical Formula: $C_{10}H_{10}N_2O_7S_2$	339
P6	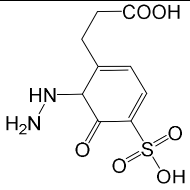 Chemical Formula: $C_9H_{12}N_2O_6S$	277
P7	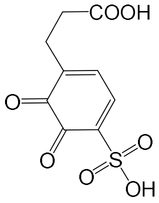 Chemical Formula: $C_9H_8O_7S$	260
P8	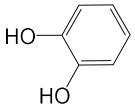 Chemical Formula: $C_6H_6O_2$	112
P9	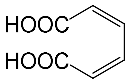 Chemical Formula: $C_6H_6O_4$	142
P10	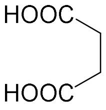 Chemical Formula: $C_4H_6O_4$	118

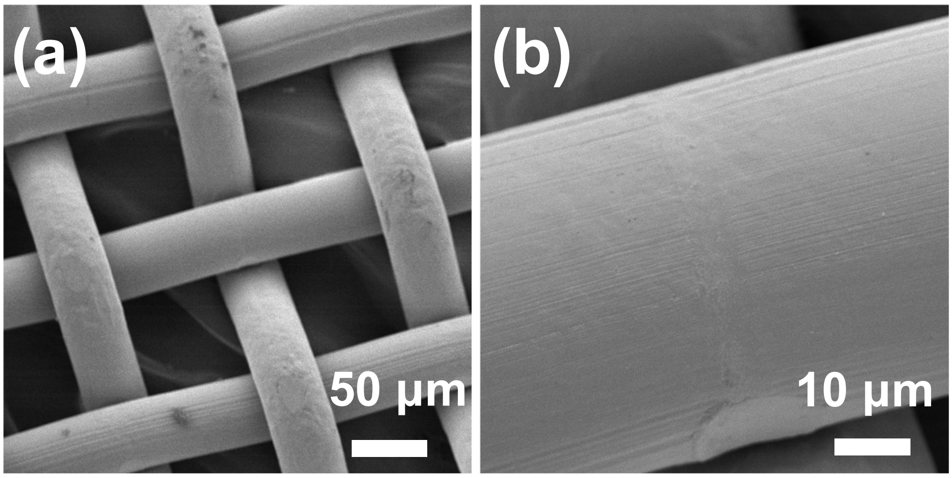

Figure S1. SEM images of the pristine Cu mesh under different magnifications.

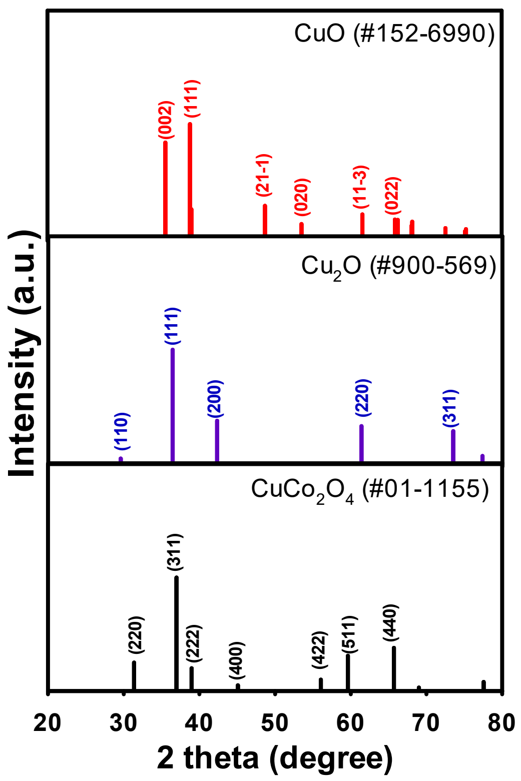

Figure S2. Simulated XRD patterns of CuO, Cu_2O and $CuCo_2O_4$.

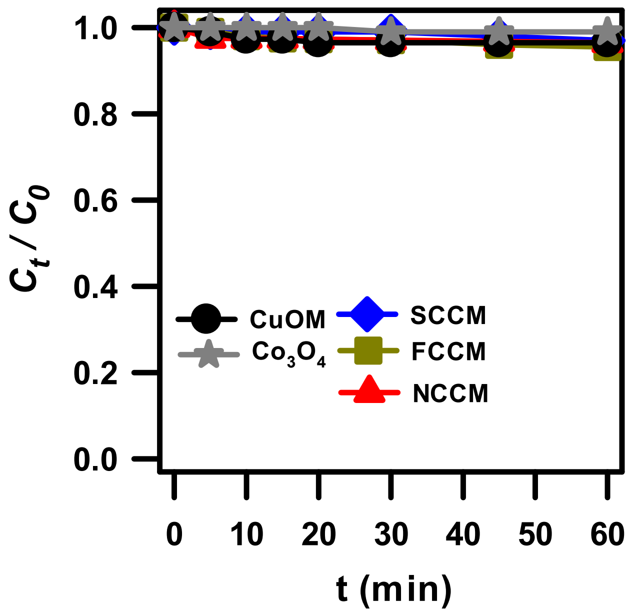

Figure S3. Removal of AR through adsorption to those catalysts (Catalyst = 200 mg/L, 30 °C).

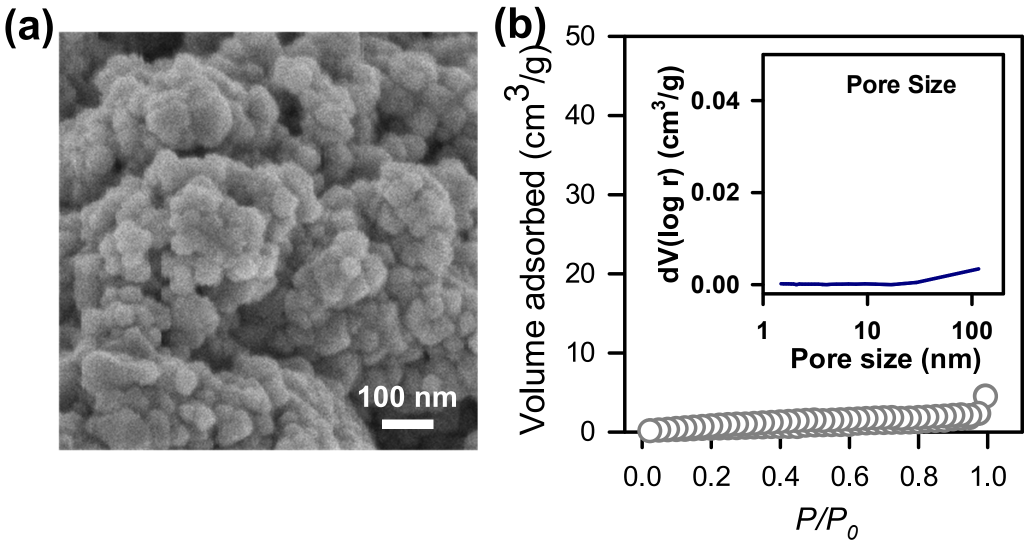

Figure S4. (a) SEM image and (b) Textural properties of the commercial Co_3O_4 NP.

Its specific surface area was determined as 2 m^2/g with a pore volume of 0.01 cm^3/g.

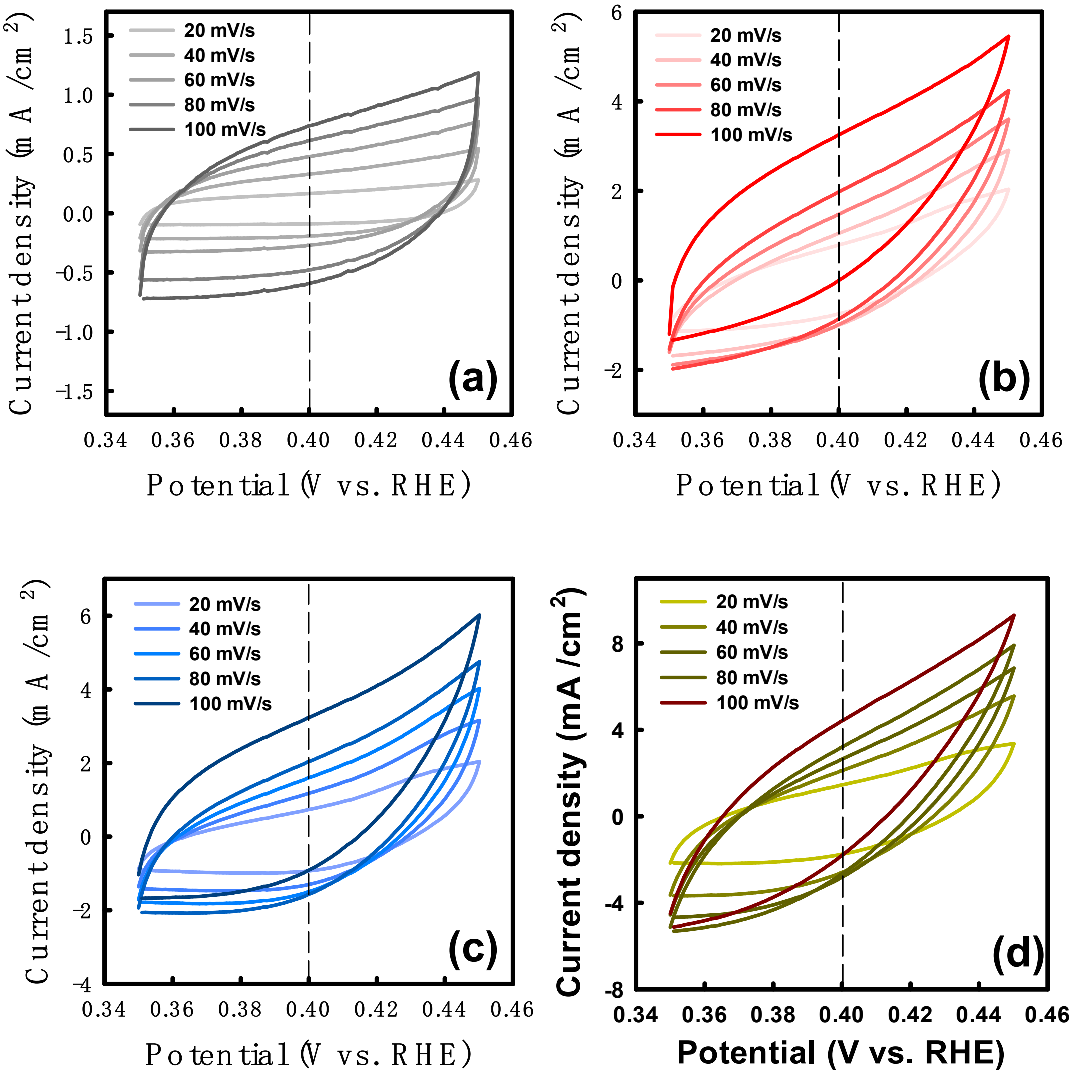

Figure S5. CV curves of (a) CuOM, (b) NCCM, (c) SCCM, and (d) FCCM at different scan rates

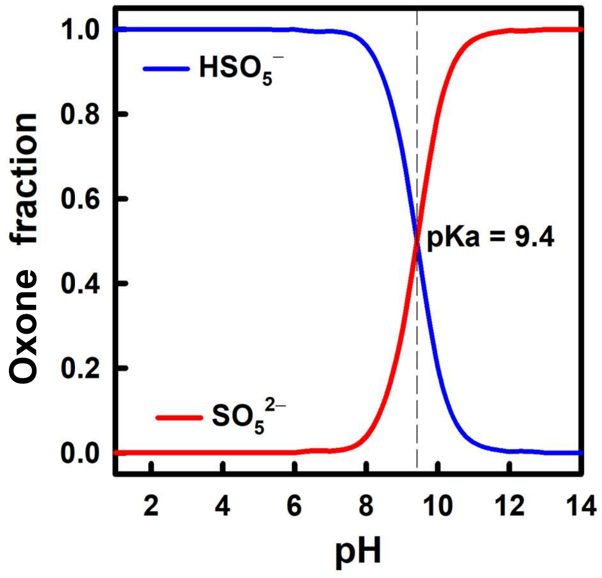

Figure S6. Species of Oxone at various pH

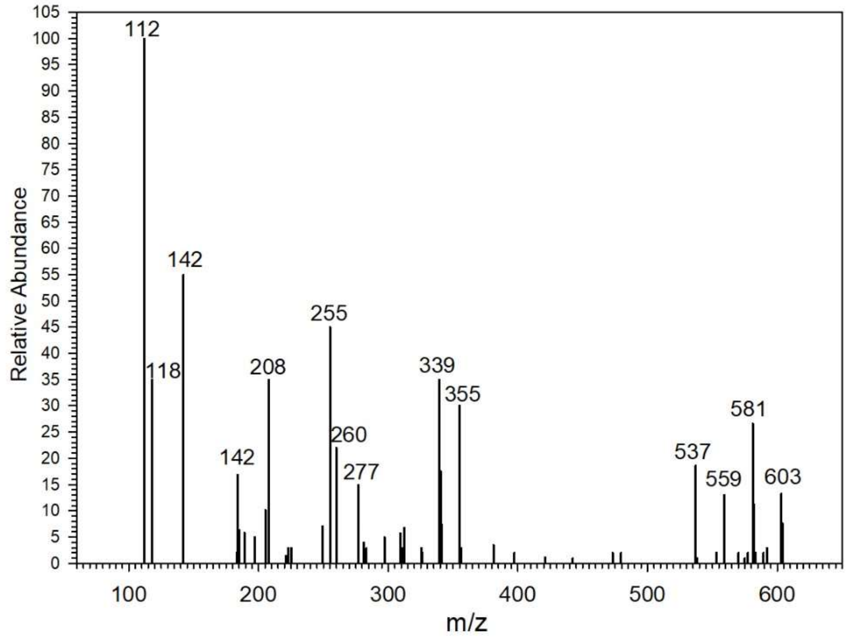

Figure S7. ESI mass spectrum of intermediates of AR degradation.

References:

[1] D.D. Tuan, W.D. Oh, F. Ghanbari, G. Lisak, S. Tong, K.-Y. Andrew Lin, Coordination polymer-derived cobalt-embedded and N/S-doped carbon nanosheet with a hexagonal core-shell nanostructure as an efficient catalyst for activation of oxone in water, J Colloid Interface Sci, 579 (2020) 109-118.
[2] K.-Y.A. Lin, Y.-C. Chen, C.-F. Huang, Magnetic carbon-supported cobalt prepared from one-step carbonization of hexacyanocobaltate as an efficient and recyclable catalyst for activating Oxone, Separation and Purification Technology, 170 (2016) 173-182.
[3] K.-Y.A. Lin, T.-Y. Lin, Degradation of Acid Azo Dyes Using Oxone Activated by Cobalt Titanate Perovskite, Water, Air, & Soil Pollution, 229:10 (2018).
[4] K.-Y.A. Lin, J.-T. Lin, X.-Y. Lu, C. Hung, Y.-F. Lin, Electrospun magnetic cobalt-embedded carbon nanofiber as a heterogeneous catalyst for activation of oxone for degradation of Amaranth dye, Journal of Colloid and Interface Science, 505 (2017) 728-735.
[5] K.A. Lin, M.T. Yang, J.T. Lin, Y. Du, Cobalt ferrite nanoparticles supported on electrospun carbon fiber as a magnetic heterogeneous catalyst for activating peroxymonosulfate, Chemosphere, 208 (2018) 502-511.
[6] M.-T. Yang, Z.-Y. Zhang, K.-Y.A. Lin, One-step fabrication of cobalt-embedded carbon nitride as a magnetic and efficient heterogeneous catalyst for activating oxone to degrade pollutants in water, Separation and Purification Technology, 210 (2019) 1-9.
[7] M.T. Yang, W.C. Tong, J. Lee, E. Kwon, K.A. Lin, CO2 as a reaction medium for pyrolysis of lignin leading to magnetic cobalt-embedded biochar as an enhanced catalyst for Oxone activation, J Colloid Interface Sci, 545 (2019) 16-24.
[8] M.-C. Li, S. Tong, J.-T. Lin, K.-Y.A. Lin, Y.-F. Lin, Electrospun Co3O4 nanofiber as an efficient heterogeneous catalyst for activating peroxymonosulfate in water, J Taiwan Inst Chem Eng, 106 (2020) 110-117.
[9] M.H. Li, K.A. Lin, M.T. Yang, B.X. Thanh, D.C.W. Tsang, Prussian Blue Analogue-derived co/fe bimetallic nanoparticles immobilized on S/N-doped carbon sheet as a magnetic heterogeneous catalyst for activating peroxymonosulfate in water, Chemosphere, 244 (2020) 125444.
[10] H.T. Nguyen, J. Lee, E. Kwon, G. Lisak, B.X. Thanh, W.D. Oh, K.-Y.A. Lin, Metal-complexed covalent organic frameworks derived N-doped carbon nanobubble–embedded cobalt nanoparticle as a magnetic and efficient catalyst for oxone activation, Journal of Colloid and Interface Science, 591 (2021) 161-172.

[11] K.-Y.A. Lin, J.-T. Lin, A.P. Jochems, Oxidation of amaranth dye by persulfate and peroxymonosulfate activated by ferrocene, Journal of Chemical Technology & Biotechnology, 92 (2017) 163-172.
